# Study on transient photocurrent induced by energy level defect of schottky diode irradiated by high power pulsed laser

**DOI:** 10.1038/s41598-023-40983-z

**Published:** 2023-09-02

**Authors:** Y. H. Wang, J. H. Su, T. W. Wang, Z. Y. Lei, Z. J. Chen, S. P. Shangguan, J. W. Han, Y. Q. Ma

**Affiliations:** 1 National Space Science Center, CAS, Beijing, China; 2 Beijing University of Posts and Telecommunications, Beijing, China; 3 College of Physics and Electronic Science, Hunan University, Changsha, China

**Keywords:** Engineering, Materials science, Physics

## Abstract

The transient photocurrent is one of the key parameters of the spatial radiation effect of photoelectric devices, and the energy level defect affects the transient photocurrent. In this paper, by studying the deep level transient spectrum of a self-designed Schottky diode, the defect properties of the interface region of the anode metal AlCu and Si caused by high-temperature annealing at 150 ℃, 200 ℃ and 300 ℃ for 1200 h have been quantitatively analyzed. The study shows that the defect is located at the position of + 0.41 eV on the valence band, the concentration is 2.8 $\times$ 10^13^/cm^2^, and the capture cross section is $\sigma$ = 8.5 $\times$ 10^17^. The impurity energy level mainly comes from the diffusion of Al atom in anode metal. We found that the defect did not cause the electrical performance degradation and obvious morphology change of the device, but the transient photocurrent increased significantly. The reason is that the high temperature treatment results in a growth in the density of states at the interface between AlCu–Si. The more mismatched dislocations and recombination center increased the reverse current of the heterojunction. The above view is proved by the TCAD simulation test.

## Introduction

Under the space radiation of different dose rates, the device will produce various problems such as disturbance, overturn, latch and even burn. At present, the reliability research of researchers mostly adopts the method of experiment or simulation^1–3^, which is based on the performance results of the device, and the mechanism research mainly focuses on the mechanism of the device structure^4, 5^. However, the research at the material level, especially the influence of the defects of semiconductor materials on the space radiation effect, is relatively insufficient. In this paper, a simple Schottky diode is designed to introduce defects and retest the transient photocurrent through high temperature treatment. Combining DLTS (Deep Level Transient Spectroscopy) technology and TCAD (Technology Computer Aided Design) simulation, the influence mechanism of energy level defects on the transient dose rate effect is studied.

## Experimental setup

### Sample preparation

**Figure 1:**
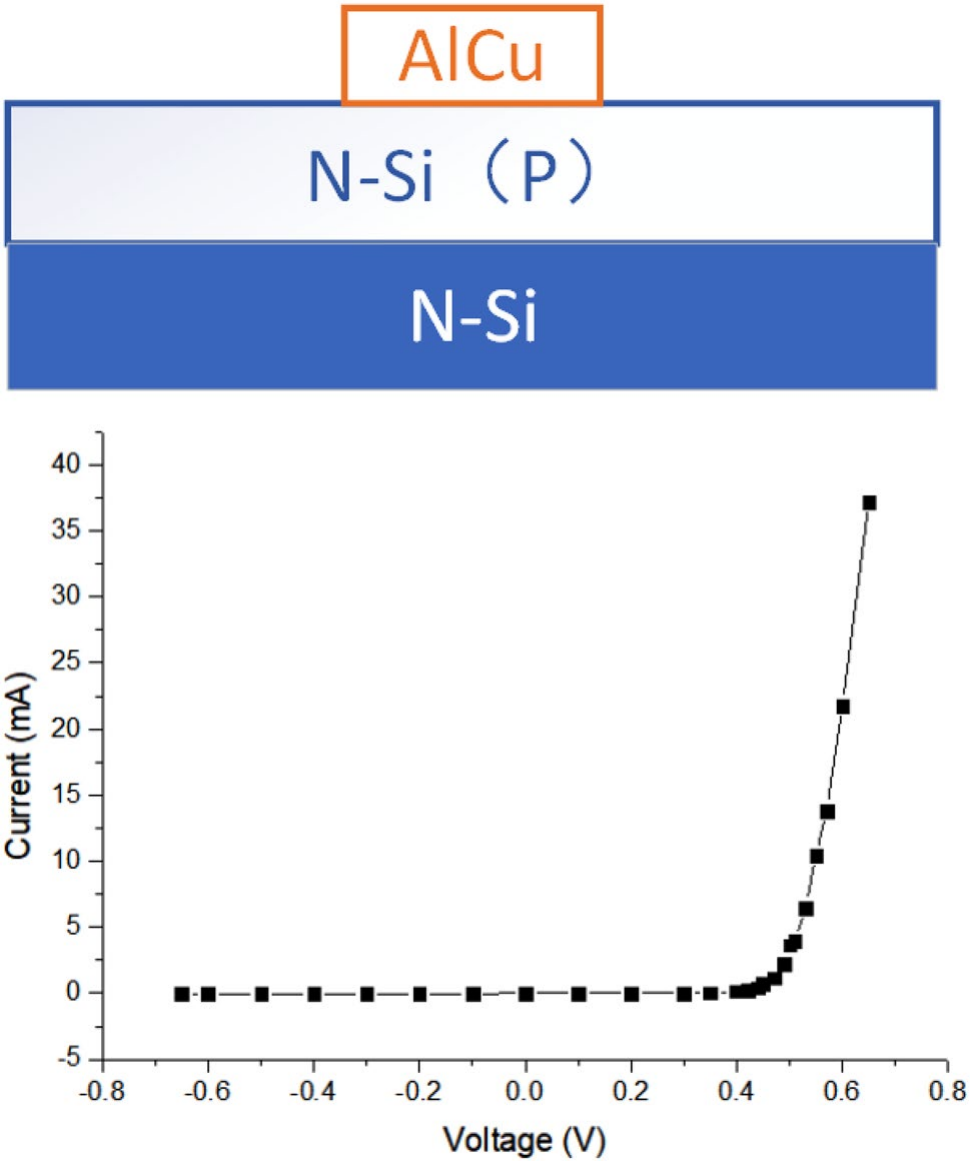
Simple Schottky diode design and I–V curve.

In order to facilitate the study on the mechanism of the influence of energy level defects on transient photocurrent, a typical Schottky diode is designed in this paper. As shown in Fig. 1, the Si substrate is N-type, (111) crystal plane, with a resistivity of 0.001–0.005 Ω cm, on which a layer of N-type Si is epitaxial grown, doped with P, with a resistivity of 2.0–2.6 Ω cm, and a doping concentration of 1.6–2.5 × 10^15^/cm^3^. The top electrode is AlCu alloy with Schottky contact. Ohmic contact shall be made on the bottom surface, and the electrode shall be paved on the bottom surface. The basic electrical properties of the fabricated device are tested by I–V curve.

### High temperature annealing

The introduction of defects through high-temperature annealing is essentially to simulate the long-term degradation of material or device performance through a short time test by introducing a temperature stress field^6^. First of all, according to different materials and devices, the temperature selected is different. For example, the white LED is 85–125 ℃^7, 8^, the GaN based device is 85 ℃^9^, the In Pb material is 150 ℃^10^, the erbium ytterbium co doped fiber amplifier is 85 ℃^11^, the organic material fluorosilicone rubber is 200 ℃^12^, the InGaAs photodiode is 100–300 ℃^13^, and the MEMS gyroscope is 125 ℃^14^. In general, the influence of high temperature treatment on devices is mainly reflected in the influence of temperature stress on electron mobility, which leads to the formation of voids or metal compounds in materials, which changes the electrical properties, the oxidation of metals caused by the presence of air oxygen, and the degradation of electrical and thermal properties caused by the denaturation of packaging materials. In this test, three ranges of 150 ℃, 200 ℃ and 300 ℃ are used for 1200 h high temperature treatment.

**Figure 2:**
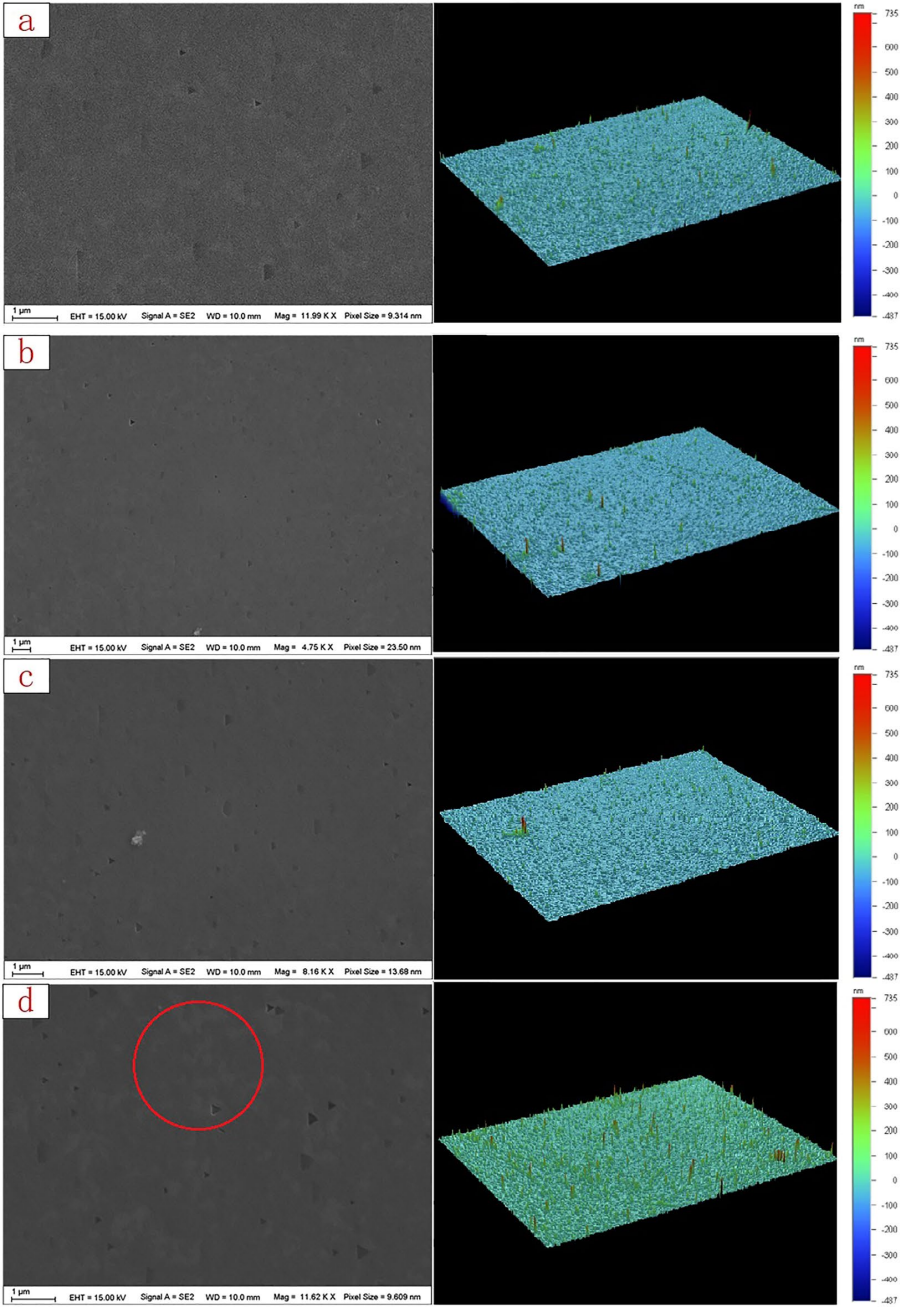
(**a**) Shows the morphology in the ground state, (**b**) shows the morphology after 150 ℃ high temperature treatment for 1200 h, (**c**) shows the morphology after 200 ℃ high temperature treatment for 1200 h, and (**d**) shows the morphology after 300 ℃ high temperature treatment for 1200 h.

**Figure 3:**
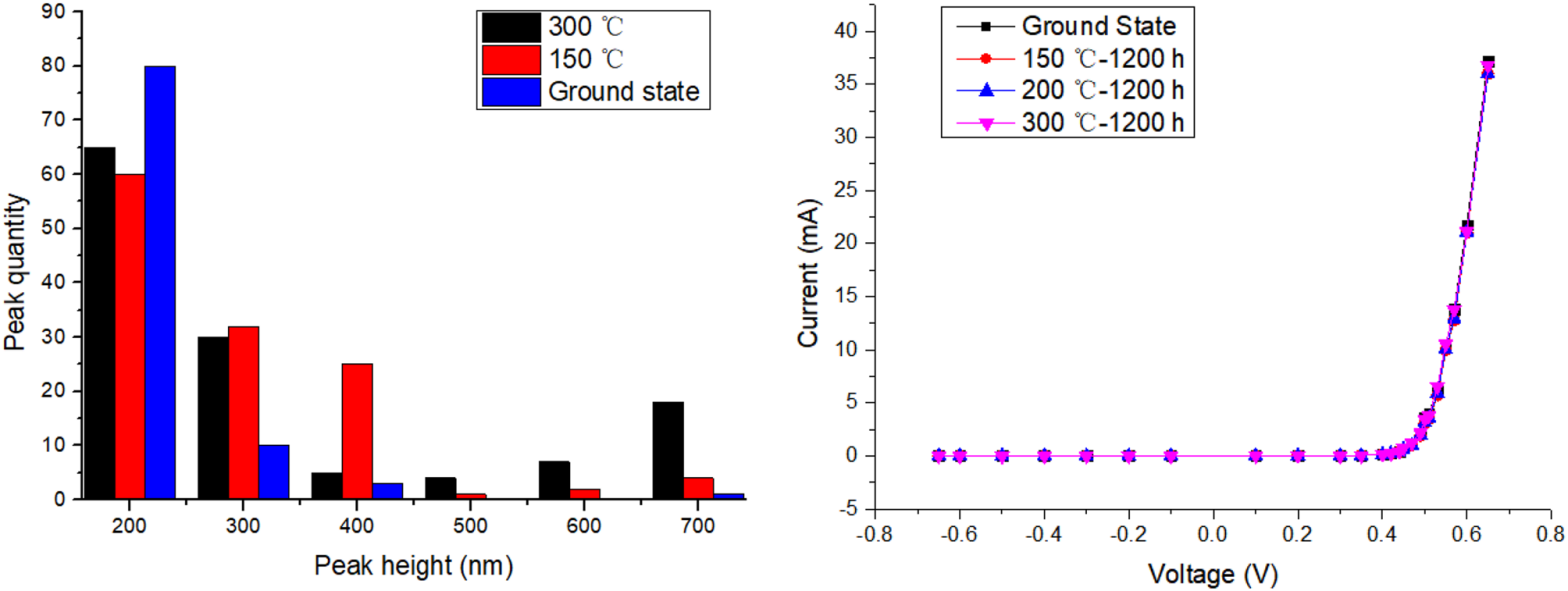
Peak quantity (left) and I–V curve (right) of device after high temperature treatment.

We used SEM (Scanning Electron Microscope) and surface morphology scanning to observe the changes of device surface morphology, as shown in Fig. 2. High-temperature annealing at 150 ℃, 200 ℃, and 300 ℃ for 1200 h is compared with the ground state, in which there is no obvious change at 150 ℃ and 200 ℃, and there are some micro visible changes at 300 ℃. The number of peaks in Fig. 3(left) explains the reason for surface morphology changes under electron microscopy. Then use the source table to test the I–V curve of the device after high-temperature treatment, as shown in Fig. 3(right).

The structure of Schottky diode is relatively simple for this test, because there is no effect on other packaging structures and materials. Generally the anode AlCu alloy used in this test has obvious oxidation phenomenon above 650 ℃ for a short time, and the structure of Schottky diode can be significantly changed after high-temperature annealing at 500 ℃ for 120 h^15^. Considering its Si substrate, the diffusion of Si atoms is obvious only when the temperature is above 900 ℃^16^. When Al Cu Si composite structure is formed, the eutectic reaction can only occur in the Al rich area at 548 °C, while no atomic diffusion phenomenon is found in the 350 °C short-term aging test^17^. Combined with the fact that there is no obvious phenomenon in the 250 ℃ aging test of InGaAs diodes with the same simple structure^13^, it can be considered that the basic electrical properties of Schottky bare chips have not been significantly affected under the aging conditions of 150 ℃, 200 ℃ and 300 ℃.

### Transient photocurrent test

**Figure 4:**
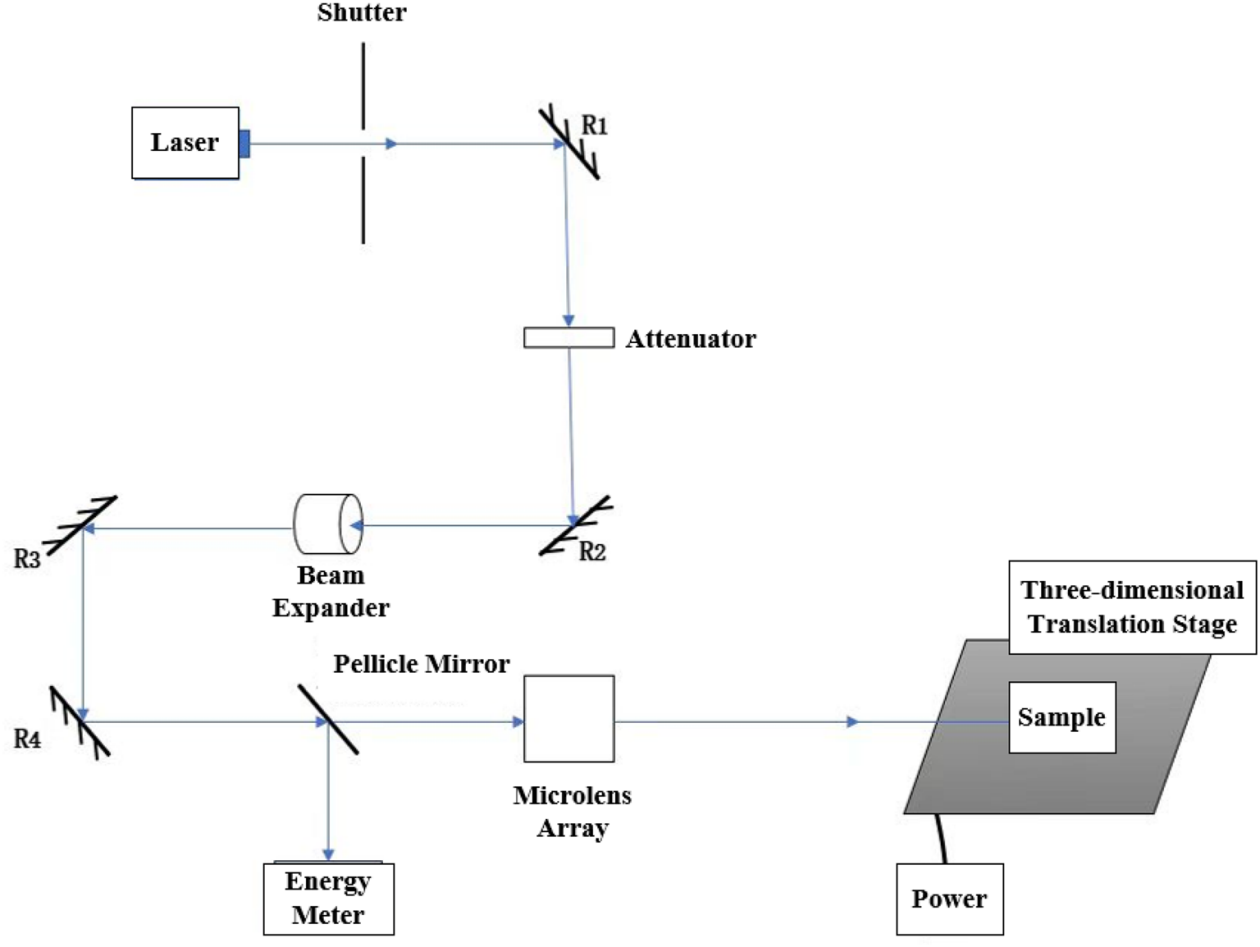
Beam path of transient photocurrent test device.

**Figure 5:**
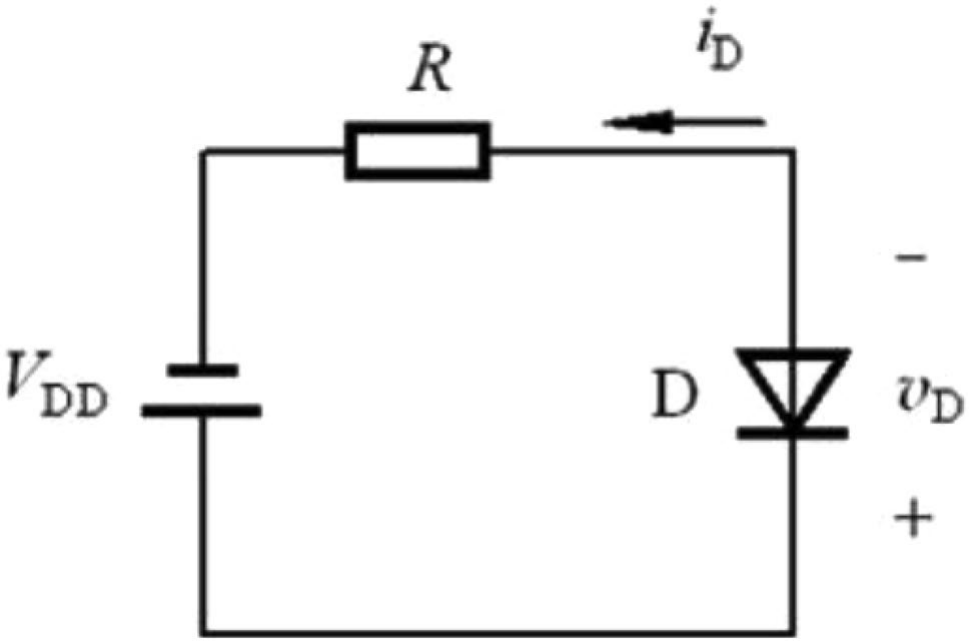
Circuit diagram of sampling resistance method.

We set up the device as shown in Fig. 4 to test the transient photocurrent of Schottky diode sample. A laser pulse from a high-energy single pulse nanosecond laser (the single pulse energy is greater than 1 J/cm^2^) first enters the beam expander through the shutter, reflector and attenuator, and expands the original 10 mm spot to 20 mm, which is convenient for full coverage of the test sample. The energy meter is used to monitor the energy of the test beam in real time, and the microlens array is used to homogenize the beam, which can improve the beam uniformity to more than 80%. At the same time, the sampling resistance method is used to test the transient photocurrent. As shown in Fig. 5, the sample is in reverse bias state, the reverse bias voltage provided by the power supply is 3 V, and the sampling resistance R = 1000 Ω.

**Figure 6:**
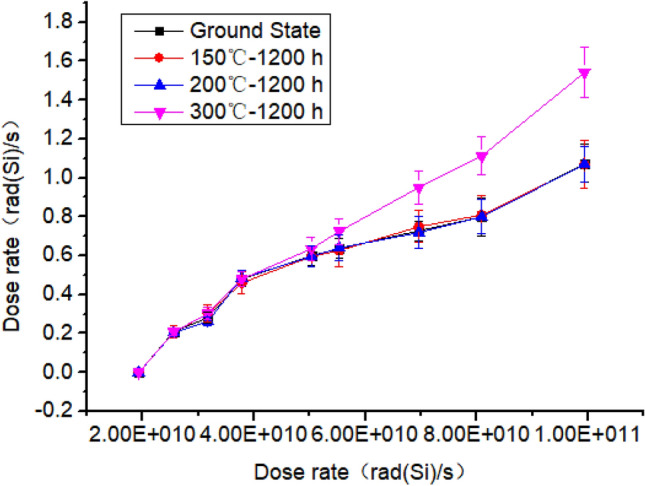
Diagram of transient photocurrent test results.

The test results of the transient dose rate are shown in Fig. 6. It is obvious that defects are introduced at 150 ℃, 200 ℃ and 300 ℃. Compared with the ground state, although the electrical performance of the device has not changed, it has significantly affected the transient photocurrent at 300 ℃, and the transient photocurrent has increased significantly.

## Discussion

**Figure 7:**
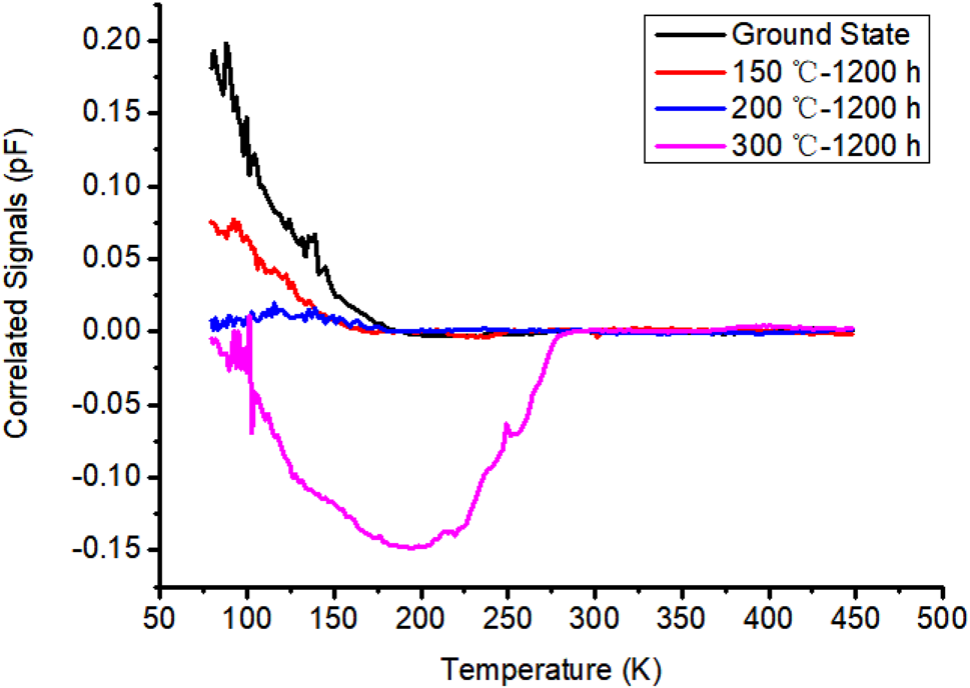
Results of DLTS deep level transient spectrum test.

To explore the causes of the above phenomena, the DLTS technology is used to test the defects of the samples as shown in Fig. 7. Under the reverse bias condition, DLTS did not detect an obvious defect peak in the samples treated with high-temperature annealing at ground state, 150 ℃ and 200 ℃, but a significant defect peak appeared in the 300 ℃ high-temperature treated samples.

It can be seen that there are slight inconspicuous multi carrier traps in the ground state, at 150 ℃ and 200 ℃, while a huge carrier trap appears at 300 ℃. The formula is used to calculate energy level status^18^:

$$e_{p,n} = \sigma_{p,n} \left\langle V \right\rangle N_{v,c} exp\left( {\frac{{ - E_{T} }}{kT}} \right)$$

$$\sigma_{p,n} = \left( {\sigma_{p,n} } \right)_{\infty } exp\left( {\frac{{ - E_{\sigma } }}{kT}} \right)$$

$$e_{p,n} = \left( {\sigma_{p,n} } \right)_{\infty } \left\langle V \right\rangle N_{v,c} exp\left( {\frac{{ - E_{Ta} }}{kT}} \right)$$

where, $E_{Ta}$ is the activation energy, $\left\langle V \right\rangle$ is the thermal motion rate of electrons, $N_{v,c}$ is the density of states, $k$ is the Boltzmann constant, and $T$ is the temperature. It can be obtained that, $E_{Ta}$ =  + 0.41 eV, the capture cross section of minority carrier trap $\sigma$ = 8.5 $\times$ 10^17^. According to the following formula,

$$N_{T} = \frac{2\Delta c\left( 0 \right)}{{c\left( \infty \right)}}\left( {N_{A} - N_{D} } \right)$$

The energy level concentration of the multi carrier defect is 2.8 $\times$ 10^13^ /cm^2^.

Generally speaking, when a short pulse laser irradiates a semiconductor material, a large number of non-equilibrium photogenerated carriers will be generated in a short time^19^. The Si–P material in this test is an N-type semiconductor, and its electrons are multi carriers, in which the minority carrier trap plays a role usually. At the same time, Si is also an indirect transition semiconductor material, which the conduction band bottom and valence band top are not at the same position in the Brillouin zone. The carrier recombination process requires assistant of phonon. The recombination center is the determinant of lifetime of minority carrier^20^. The minority carrier trap introduced by high temperature treatment promotes the recombination of unbalanced carriers, shortens the lifetime of unbalanced carriers and improved the transient photocurrent.

It is speculated that the change of interface state between AlCu Si leads to the generation of defects. The interface state is a heterojunction formed near the interface when two different materials contact. The interface maintains lattice continuity, but a large number of interface states or dangling bonds are generated on the interface. Generally, the greater the density of interface states, the greater the lattice mismatch, and the more mismatched dislocations^21^. Dislocation defects play the role of recombination center, which will reduce minority carrier lifetime and minority carrier diffusion length. For heterostructures, the reverse current is significantly increased^22^.

**Figure 8:**
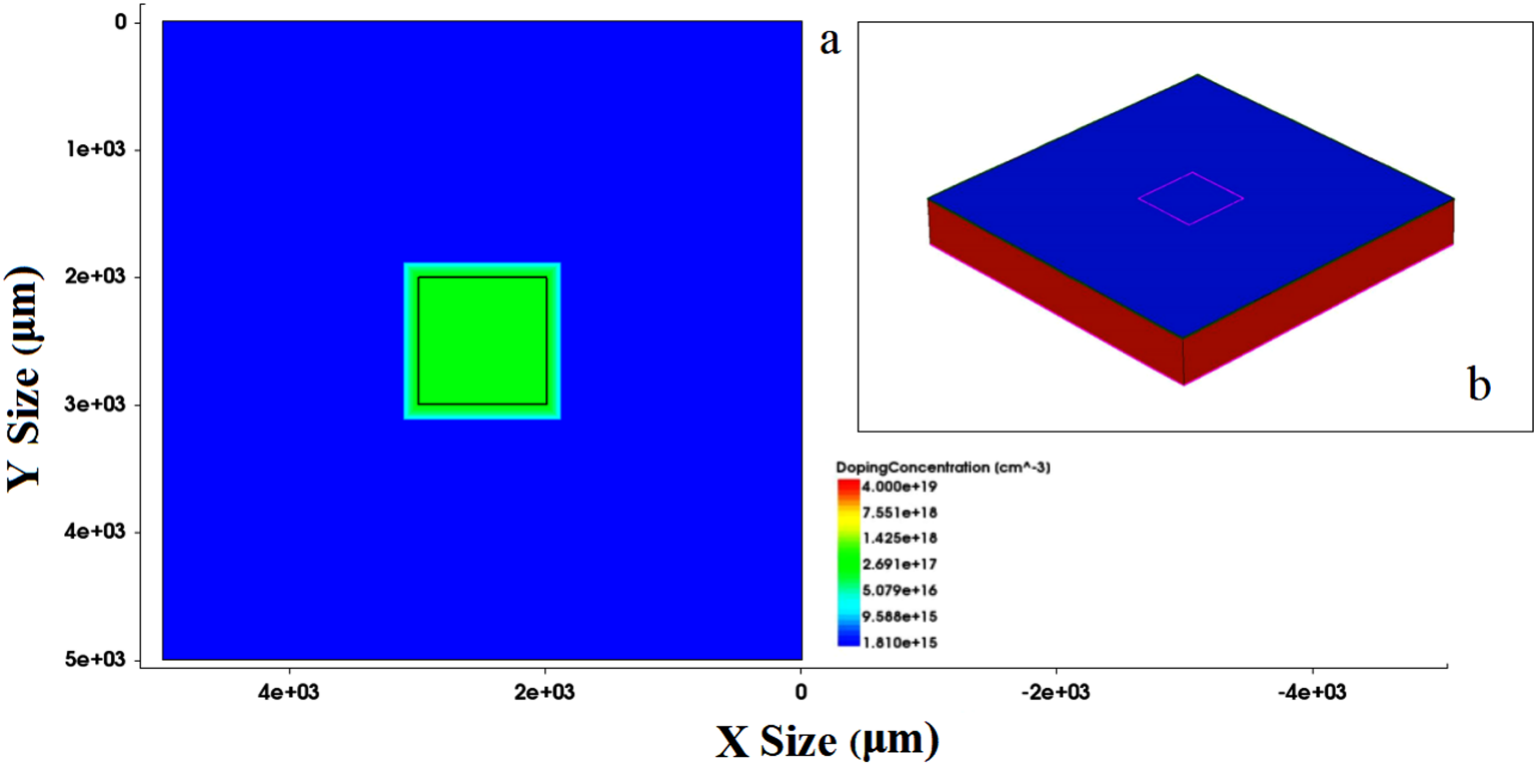
TCAD simulation modeling structure diagram.

**Figure 9:**
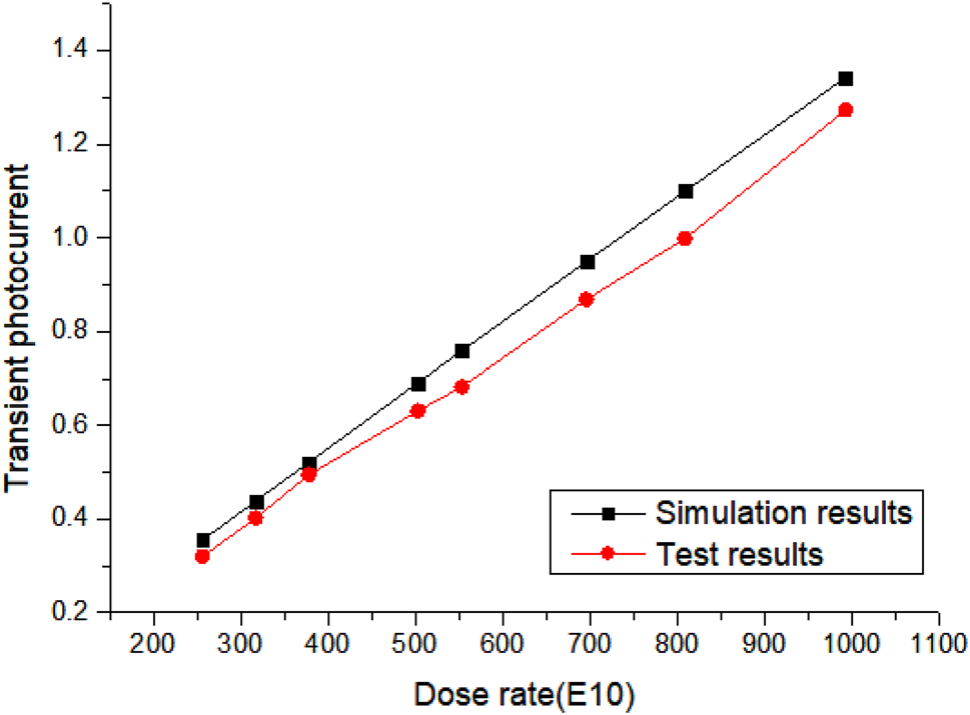
Deviation between TCAD simulation and actual measurement.

TCAD simulation is used to model the device to prove the above assumption as shown in Fig. 8 (Fig. 8b is a three-dimensional display, not applicable to coordinate axis dimensions). The validity of the model is proved by comparing the simulated IV curve with the measured IV curve. As shown in Fig. 9, the deviation of the simulated and measured current under the same voltage is less than 20% on average.

**Figure 10:**
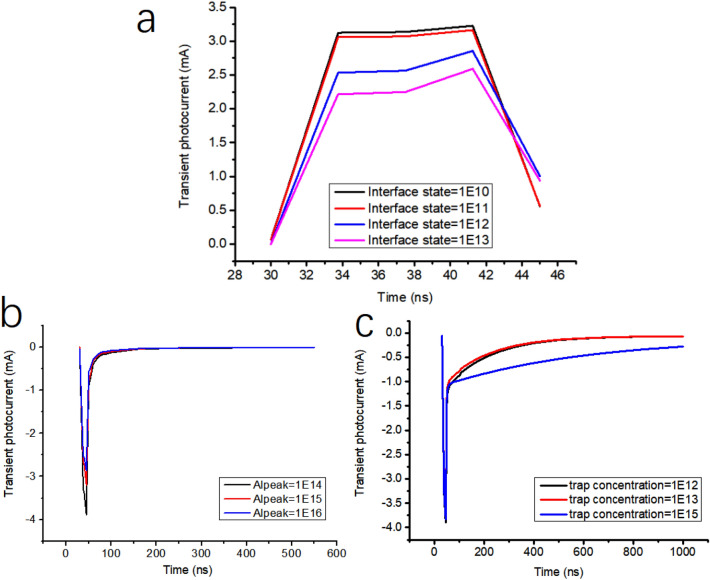
TCAD simulation interface state density (**a**), aluminum atom diffusion (**b**) and trap concentration (**c**) affect transient photocurrent. All units in the figure are particle/cm^2^.

**Figure 11:**
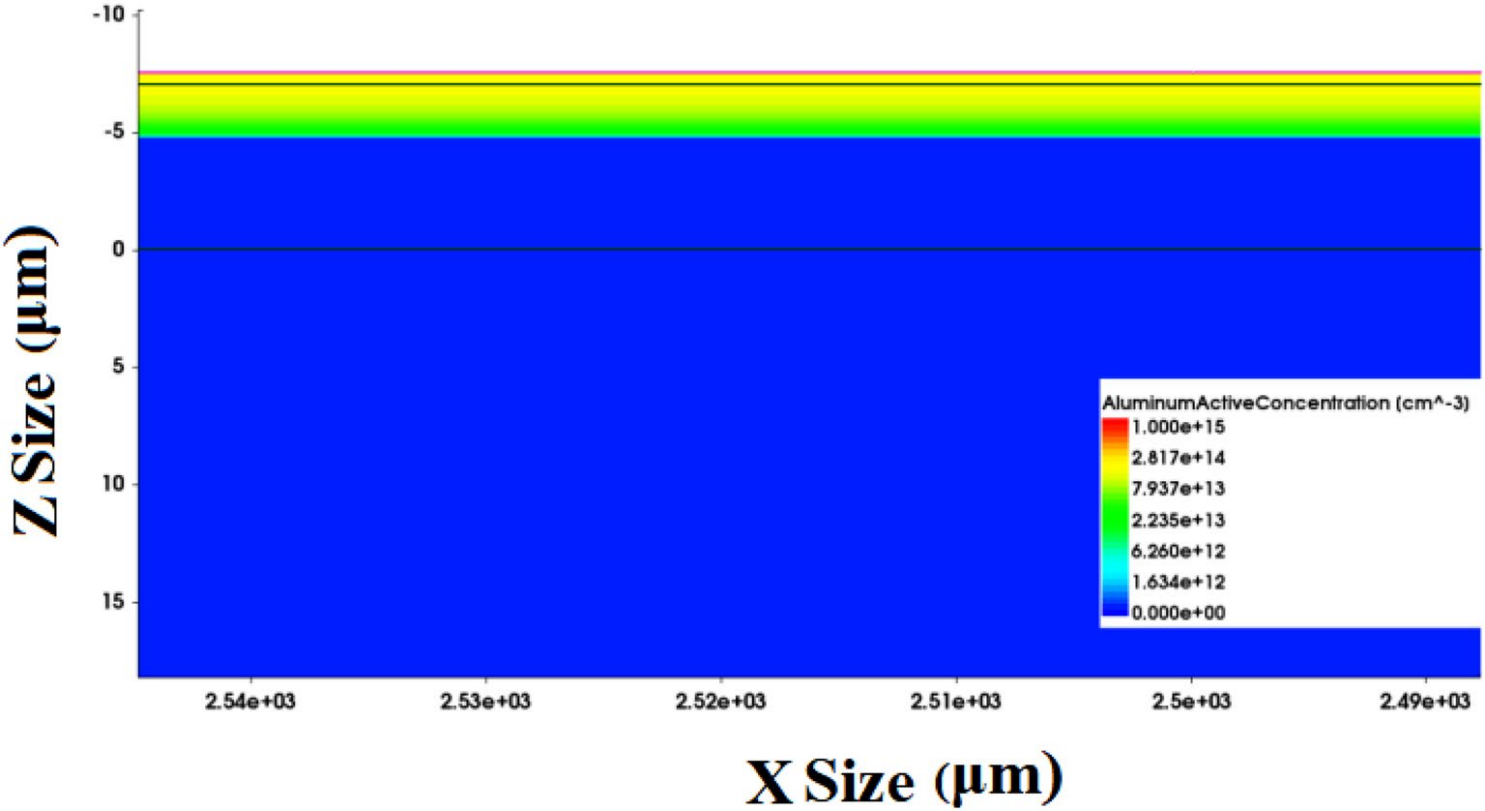
Device profile with interface state density 1 $\times$ 10^13^/cm^2^.

The model is used to study the influence of interface state density on the transient photocurrent. As shown in Figs. 10 and 11, TCAD simulates that interface state density, aluminum atom diffusion and trap concentration affect the transient photocurrent. The interface state density increases from 1 $\times$ 10^10^/cm^2^ to 1 $\times$ 10^13^/cm^2^ as shown in Fig. 10a. It can be seen that with the increase of interface state density, the transient photocurrent has an obvious downward trend, which is consistent with the previous test results (Due to the reverse bias condition during transient photocurrent testing, the positive current during simulation is called negative current during testing). The increase in trap concentration in the body region leads to a significant decrease in transient photocurrent. Figure 10b and c show a strong correlation between the diffusion of aluminum atoms and the effect of trap concentration on transient photocurrent, which is due to the increase in trap concentration and interface density of states caused by aluminum atom diffusion. Among them, the diffusion of aluminum atoms mainly afftects the peak of transient photocurrent, while the concentration of bulk defects shows a certain degree of influence in the long term. The influence of aluminum atom diffusion and bulk defect density is different, and the reasons for this need to be further studied.

## Conclusions

DLTS and TCAD simulation technology are used to prove that the influence mechanism of defects introduced by high temperature annealing treatment do not lead to changes in basic electrical properties on transient photocurrent. It is that the interface state formed between AlCu-Si is a minority carrier trap defect, which reduces minority carrier lifetime and leads to the improve of transient photocurrent. Therefore, some deep-level defects of materials cannot be detected by general electrical performance tests, which lead to unknown devices problems for the scene requiring high reliability (such as space radiation effects). In this paper, the deep level transient spectrum defect detection technology may be used as a test method for the reinforcement of space radiation effect.

## Data Availability

The datasets generated and/or analysed during the current study are not publicly available, because the samples and laser experimental setup involve special test items which is not convenient for disclosure. However, these are available from the corresponding author on reasonable request.
